# Vacuum 5‐step technique versus OdonAssist: Which is easier to learn for professionals without experience in assisted vaginal births? A simulation study

**DOI:** 10.1002/ijgo.70836

**Published:** 2026-01-28

**Authors:** Marcos Javier Cuerva, Candela Durá, Andrés Aramendia, Marta Cortes, Francisco Lopez, José Luis Bartha

**Affiliations:** ^1^ Department of Obstetrics Hospital Universitario La Paz Madrid Spain; ^2^ Facultad de Medicina Universidad Autónoma de Madrid Madrid Spain; ^3^ Facultad de Ciencias Biomédicas y de la Salud Universidad Alfonso X el Sabio (UAX) Madrid Spain

**Keywords:** assisted vaginal birth, OdonAssist, operative birth, simulation training, vacuum

## Abstract

**Objective:**

To compare the ease of learning the Vacca 5‐step vacuum technique versus the OdonAssist device for assisted vaginal birth (AVB) among healthcare professionals without previous experience in AVBs.

**Methods:**

A prospective, randomized crossover, simulation‐based study was conducted with 12 final‐year medical students. Each participant underwent training in both techniques. The primary outcome was the number of attempts required to achieve three consecutive error‐free AVBs on a simulation model. Secondary outcomes included procedure duration, perceived difficulty, and error rates.

**Results:**

A total of 24 training sessions were studied, including 123 simulated AVBs (74 vacuum, 49 OdonAssist). Participants required fewer attempts to master the OdonAssist technique compared with the Vacca 5‐step vacuum technique (4, interquartile range (IQR) 3–5, attempts versus 6, IQR 4–8, attempts; *P* = 0.012). Training sessions and AVBs with OdonAssist were also perceived as easier.

**Conclusion:**

Both techniques can be effectively taught using simulation‐based methods. However, the OdonAssist device was easier to learn and required fewer repetitions, making it a promising tool for introducing AVB into settings where AVB is underused.

## 1 INTRODUCTION

Assisted vaginal birth (AVB) is an important second‐stage obstetric intervention. Despite its benefits in reducing maternal and neonatal morbidity, AVB use has declined globally, with rates below 10% in most low‐ and middle‐income countries and as low as 1% in some African and American nations.^1^ Although prevention of primary cesarean section primarily depends on appropriate labor management, AVB remains a valuable option to avoid cesarean delivery in selected cases.^2^ Its underutilization reflects persistent systemic barriers, including inadequate training, fear of litigation, and limited institutional support.^3^, ^4^

Among AVB instruments, the vacuum extractor is currently the most widely used worldwide.^5^ The “5‐step technique” developed by Aldo Vacca is widely accepted, especially with maneuverable vacuum devices.^6^ This technique emphasizes accurate identification of the fetal head flexion point, controlled cup insertion, and synchronized traction with uterine contractions. It has shown superior accuracy in simulator‐based studies, particularly in challenging fetal head positions such as posterior and transverse.^7^

Recently, a novel device employing a standardized technique, the OdonAssist®, has introduced a new alternative for AVB. The OdonAssist is a single‐use device that received regulatory approval within the European Union in 2025. OdonAssist uses an inflatable polymer sleeve that grips the fetal head. Early clinical evaluations report favorable safety and feasibility outcomes.^8^, ^9^, ^10^, ^11^

This study arises from the need to improve training in AVB techniques, particularly in settings where AVB is rarely practiced. To address this gap, the aim of the present study was to evaluate which instrument and technique (vacuum extraction using the five‐step technique or the OdonAssist device) is more easily learned by healthcare professionals with no experience in AVB, using a simulation‐based training model.

## 2 MATERIALS AND METHODS

This was a simulation‐based, experimental, prospective, non‐blinded, randomized crossover study conducted between September and October 2025. The institutional board approved the study, and the Ethics Committee of Hospital Universitario La Paz granted an exemption from approval, as no health data or biologic samples were involved (Acta No. 17/2025; certificate issued September 24, 2025; reference 2025.714). As no patients participated, informed consent from patients was unnecessary. Informed consent was obtained from all study participants.

This experimental study included 24 independent training sessions involving 12 final‐year medical students who had already completed and passed the obstetrics and gynecology course. All participating students received training in both AVB techniques (vacuum extraction using the five‐step technique and OdonAssist) using a randomized crossover design. The students were randomly assigned to the order in which the two techniques were taught, performing either vacuum extraction or OdonAssist training first.

Each training session was conducted with a 1:1 trainer to student ratio. The trainers (MJC or MC) are certified instructors in simulation training, as well as in both the Vacca 5‐step technique and OdonAssist AVB. During each simulation, a research assistant (CD or AA) collected the study data.

For the training sessions, the Peyton methodology was followed, consisting of four steps: Demonstration, Deconstruction, Comprehension, and Performance.^12^ In the fourth step, two AVBs were performed. After completing the four steps, the study phase began, during which participants performed simulated AVBs until they achieved three consecutive error‐free procedures. During the study phase, participants only received feedback after the simulation and only if they had made an error. Throughout the entire process, the sequence of fetal head positions was: occiput anterior, occiput posterior, and then occiput transverse.

The simulations for vacuum AVB were conducted using the Lucy and Lucy's Mum Instrumental Delivery Birth Simulator (MODEL‐med^®^, Australia). This simulator is also the one used in training courses offered by the Vacca Academy. For the procedures, the Kiwi Omnicup^®^ (Laborie medical technologies corp., USA) was chosen as the vacuum instrument. For the OdonAssist simulations, a PROMPT Flex Advanced Birthing Simulator (Limbs & Things Ltd., UK) equipped with the 80 310 AVB module was used, together with OdonAssist devices (Maternal Newborn Health Innovations, PBC, USA). This is the same simulator employed by Maternal Newborn Health Innovations, PBC, in their official OdonAssist training courses.

The Vacca 5‐step technique for vacuum AVB is a five‐step process that involves the following steps: (1) identify the location of the flexion point and determine its distance from the maternal posterior fourchette; (2) insert the cup into the introitus; (3) maneuver the cup posteriorly along the maternal pelvis axis until the measured distance in step one is reached, which can be verified by checking the distance marks on the suction tubing; (4) create the necessary vacuum and ensure the exclusion of maternal tissue; and (5) provide traction along the axis of the maternal pelvis (Video S1).

The OdonAssist procedure follows a standardized technique: (1) first, during the preparation phase, the four inner channels, sleeve, and cup are thoroughly lubricated; (2) the application step begins with placing the cup on the fetal head, then removing the fastening band; (3) during a uterine contraction, the sleeve is advanced into the birth canal until the number “0” appears in the viewing window; (4) the device is inflated by pumping eight times; (5) the inserter is removed, and the pump is activated two more times; (6) the operator grasps both handles and, following the curve of the birth canal, applies progressive traction during uterine contractions; (7) the operator deflates the device as the fetal head crowns using the deflation button; (8) the deflation button is held down, the handles are maintained with one hand while the other hand provides perineal support, and the birth is completed (Video S2).

The data collected during each training session included: participant age, university, nationality, dominant hand, self‐identified gender, glove size, technique learned, technique learned first according to randomization, number of attempts until achieving three consecutive error‐free AVBs, fetal position in each AVB, presence or absence of errors, steps in which errors occurred (if any), total time for each AVB (in seconds), perceived difficulty of each AVB, total duration of the training session (in minutes), perceived difficulty of the training session, and total number of errors during the training session.

Errors were defined as incorrect or omitted steps in the techniques. In AVBs using vacuum, a mistake in step 3 was considered any cup placement with a displacement greater than 1 cm from the flexion point, either laterally or vertically.^7^

Perceived difficulty was assessed using a five‐point Likert scale, where one corresponded to “very easy”, two to “easy”, three to “moderate”, four to “difficult”, and five to “very difficult”.

The sample size was calculated based on data from previous simulation‐based training conducted with obstetrics and gynecology residents to teach the Vacca 5‐step technique. Using the described teaching methodology, participants required an average of 6.2 attempts, with a standard deviation of 1.73, to successfully perform three consecutive AVBs without errors. To detect a minimum difference of two simulated AVBs between techniques, assuming a common standard deviation of 1.73, with a two‐sided significance level (α) of 0.05 and a statistical power of 80% (1 – β), 12 participants were required (a total of 24 training sessions).

Numerical variables were expressed as median (interquartile range (IQR)) and qualitative variables as absolute and relative frequencies.

Training session level outcomes (training time, procedures required to achieve three consecutive successful attempts, total errors, and perceived difficulty) were compared between techniques using paired nonparametric tests (Wilcoxon signed‐rank test).

Case‐level outcomes were analyzed using linear mixed effects models with a random intercept for operator to account for repeated procedures within operators. Fixed effects included technique, training order (technique learned first), case number (to account for learning effects), and fetal head position. Clinically relevant interaction terms were evaluated to assess potential effect modification, including interactions between technique and training order, technique and case number, and technique and fetal head position.

The level of significance was set at 0.05. All analyses were performed using SPSS version 28.0 (IBM, Armonk, NY, USA).

## 3 RESULTS

A total of 24 training sessions were studied, including 123 simulated AVBs. The duration of each training session was 24 (IQR 21.5–30.5) minutes. There were 74 vacuum and 49 OdonAssist AVBs. The participants were 23 (IQR 22–23) years old, all right‐handed; 9 (75%) identified as women and 3 (25%) as men. They came from four different universities: 6 (50%) from Universidad Autónoma de Madrid, 4 (33.3%) from Universidad Alfonso X el Sabio, 1 (8.3%) from Universidad de Navarra, and 1 (8.3%) from Cardiff University. Regarding nationality, 10 (83.3%) were Spanish, 1 (8.3%) was from Venezuela, and 1 (8.3%) was from Wales. As for glove size, 7 (58.3%) used size 6.5, 4 (33.3%) used size 7, and 1 (8.3%) used size 7.5.

At the training session level, training in vacuum AVB using the Vacca 5‐step technique required a significantly greater number of simulated procedures than training with OdonAssist, with medians of 6 (IQR 4–8) and 4 (IQR 3–5), respectively (*Z* = −2.51, *P* = 0.012). Total training duration was longer for vacuum AVB than for OdonAssist, without reaching statistical significance (*Z* = −1.77, *P* = 0.077). Perceived difficulty of the training session was higher for vacuum AVB compared with OdonAssist (3 (IQR 2–4) versus 2 (IQR 2–2); *Z* = −2.55, *P* = 0.011). The total number of errors per training session was also higher for vacuum AVB (1.5 (IQR 0.25–3.0) versus 1.0 (IQR 0.0–1.0); *Z* = −1.99, *P* = 0.046) (Table 1).

**TABLE 1:** Training‐level outcomes: Comparison between the five‐step vacuum technique and OdonAssist.^a^

	Vacuum training (*n* = 12)	OdonAssist training (*n* = 12)	Wilcoxon *Z*	*P* value	Effect size *r*
Attempts to achieve three error‐free procedures	6 (4–8)	4 (3–5)	−2.51	0.012	0.72
Training session duration, min	28.5 (22.5–32.5)	24.0 (20.5–27.0)	−1.77	0.077	0.51
Overall perceived difficulty of training (1–5)	3 (2–4)	2 (2–2)	−2.55	0.011	0.74
Total number of errors	1.5 (0.5–3.0)	1.0 (0.0–1.0)	−1.99	0.046	0.57

^a^ Values are expressed as median (interquartile range). Paired comparisons were performed using the Wilcoxon signed‐rank test. Effect size *r* was calculated as |*Z*|/√*N*.

At the case level, vacuum AVBs were completed in a shorter time than OdonAssist AVBs, with a procedure duration of 96 (IQR 64–121) versus 117 (IQR 97–137) seconds (Table 2). Despite this unadjusted difference, mixed‐effects analyses accounting for repeated procedures within operators and training‐related factors showed that procedure duration was not independently associated with technique (Table S1).

**TABLE 2:** Case‐level procedure duration and perceived difficulty by technique.^a^

	Vacuum AVBs (*n* = 74)	OdonAssist AVBs (*n* = 49)
Procedure duration, s	96 (64–121)	117 (97–137)
Perceived difficulty (1–5)	3 (2–4)	2 (2–2)

Abbreviation: AVB, assisted vaginal birth.

^a^ Values are median (interquartile range).

Perceived difficulty at the case level was higher for vacuum AVBs compared with OdonAssist AVBs, with scores of 3 (IQR 2–4) and 2 (IQR 2–2) (Table 2). However, perceived difficulty decreased significantly with increasing case number, indicating a learning effect (Table S2).

When stratified by fetal head position, no clinically relevant or statistically significant differences in procedure time or perceived difficulty were observed within either technique (Tables 3 and 4).

**TABLE 3:** Case‐level outcomes by fetal head position in vacuum assisted vaginal births.^a^

	Occiput anterior (*n* = 26)	Occiput posterior (*n* = 25)	Occiput transverse (*n* = 23)
Procedure duration, s	96 (60–121)	105 (67–124)	95 (65–120)
Perceived difficulty (1–5)	3 (1–3)	3 (3–4)	3 (3–4)

^a^ Values are median (interquartile range).

**TABLE 4:** Case‐level outcomes by fetal head position in OdonAssist assisted vaginal births.^a^

	Occiput anterior (*n* = 20)	Occiput posterior (*n* = 16)	Occiput transverse (*n* = 13)
Procedure duration, s	122.5 (100.5–149)	122.5 (97.5–141)	107 (84–114)
Perceived difficulty (1–5)	2 (2–2)	2 (1–2)	2 (1–2)

^a^ Values are median (interquartile range).

A total of 24 mistakes were observed among the 21 vacuum AVBs with errors, and 10 mistakes among the 10 OdonAssist AVBs with errors. Of the vacuum AVB errors, 20 (83.3%) occurred during step 3, as the center of the cup was not optimally positioned in relation to the flexion point. For the OdonAssist, the most common error occurred four times (40%) and involved holding the sleeve while applying the OdonAssist.

Descriptive analyses did not reveal any clinically meaningful differences in training or case‐level outcomes across operator characteristics, such as gender, glove size, nationality, or university.

## 4 DISCUSSION

Among potential users with no experience in AVB, our data show that learning the OdonAssist technique is perceived as easier and requires fewer repetitions compared with learning the Vacca 5‐step technique for vacuum AVB. Considering that AVBs are not being performed in many settings, and that there is a growing need to begin their implementation,^1^ OdonAssist appears to be a potentially useful tool for this purpose.

One of the main barriers to the implementation of AVBs is the complexity of training. The introduction of standardized techniques, such as the Vacca 5‐step technique, represented a significant advancement by simplifying the learning process. In fact, the adoption of this technique alongside the Kiwi Omnicup device has led to a reduction in the use of obstetric forceps in several countries.^13^, ^14^ Currently, the incorporation of the OdonAssist device, which also employs a standardized technique, appears to follow a similar path in facilitating both the implementation and learning of AVB.^10^ In our study, we found that through an evidence‐based teaching methodology and a 1:1 instructor to trainee ratio,^12^ both the five‐step technique for vacuum AVB and the OdonAssist technique can be effectively taught in approximately 25–30 minutes.

One aspect that stands out in favor of OdonAssist in the literature is that its technique does not require modification based on fetal head position.^9^, ^10^, ^11^ However, the Vacca 5‐step technique also does not require changes in technique depending on fetal head position but rather relies on accurate identification of the flexion point to ensure optimal cup placement. In this context, intrapartum ultrasound, which allows for reliable determination of fetal head position before AVB, has proven to be useful and has improved outcomes in AVBs.^15^ In our study, a known sequence of fetal head positions was followed, meaning that participants were certain of the position, similar to clinical practice when intrapartum ultrasound is used. This may explain why no differences were observed in the use of the Vacca 5‐step technique across different fetal head positions. However, it is important to highlight that the most common error during vacuum AVBs occurred in step 3 (“maneuver the cup posteriorly along the maternal pelvis axis until the measured distance in step one is reached”), where the cup was incorrectly positioned. This suggests that although the vacuum technique itself remains the same, the need to adjust the cup's displacement distance depending on each individual case may explain why participants found the use of OdonAssist easier.

Another relevant finding was that randomization confirmed the stability of the results, regardless of whether participants had previously learned an alternative technique. Participants consistently rated the OdonAssist as easier to learn, independent of previous training with the Vacca 5‐step technique. On the other hand, it is important to consider whether both simulators offered equally suitable conditions for their use. Although two different simulators were employed, each was selected according to internationally recognized recommendations for its respective instrument; therefore, each can be regarded as the most appropriate for the corresponding device.^16^, ^17^, ^18^

Regarding strengths and limitations, a key strength of this study is the inclusion of final‐year medical students, ensuring minimal previous experience with AVB techniques and reducing bias from previously acquired procedural skills, thereby allowing a more uniform comparison across techniques. However, the use of students may also represent a limitation, as their high motivation could have contributed to shorter procedural times. In addition, the simulation‐based design may underestimate procedure duration by excluding physiologic variables such as synchronization with uterine contractions.^19^ Finally, all predefined technical errors were weighted equally to focus on technical learnability rather than clinical risk; nevertheless, certain errors associated with vacuum extraction, particularly incorrect cup placement, may carry greater maternal and neonatal risk in clinical practice.^8^ This limitation should be considered when interpreting the results and extrapolating them to real‐world settings.

In conclusion, although both the Vacca 5‐Step and OdonAssist techniques can be effectively taught in a short period of time using evidence‐based training methods, the OdonAssist technique stands out as a particularly promising option for AVB training among users with no experience. Its perceived ease of learning, reduced need for repetition, and standardized approach, unaffected by fetal head position, highlight its potential as a valuable tool for promoting the global implementation of AVB.

## AUTHOR CONTRIBUTIONS

MJC contributed to protocol/project development, data collection, data analysis, and manuscript writing; CD contributed to protocol/project development, data analysis, and manuscript writing; AA contributed to data collection and manuscript editing; MC contributed to protocol/project development, data collection, and manuscript editing; FL contributed to data analysis and manuscript writing/editing; and JLB contributed to protocol/project development, validation of data analysis, and manuscript reviewing and editing. All authors contributed to editorial changes in the manuscript. All authors read and approved the final manuscript. All authors have participated sufficiently in the work and agreed to be accountable for all aspects of the work.

## FUNDING INFORMATION

This research did not receive any specific grant from funding agencies in the public, commercial, or non‐profit sectors.

## CONFLICT OF INTEREST STATEMENT

The authors have no conflicts of interest.

## Supporting information


**Table S1.** Linear mixed‐effects model for procedure duration at the case level.

**Table S2.** Linear mixed‐effects model for perceived difficulty at the case level.

**Video S1.** Vacca 5‐step technique on the simulation model used in the study.

**Video S2.** OdonAssist technique on the simulation model used in the study.

## Data Availability

The data that support the findings of this study are available from the corresponding author upon reasonable request.

## References

[1] Ubom AE , Barnea ER , DiSimone N , et al. FIGO good practice recommendations: assisted vaginal birth and the second stage of labor. Int J Gynaecol Obstet. 2025;171(3):970‐982. doi:10.1002/ijgo.70528 40994258

[2] American College of Obstetricians and Gynecologists (College); Society for Maternal‐Fetal Medicine , Caughey AB , Cahill AG , Guise JM , Rouse DJ . Safe prevention of the primary cesarean delivery. Am J Obstet Gynecol. 2014;210(3):179‐193. doi:10.1016/j.ajog.2014.01.026 24565430

[3] Harrison MS , Saleem S , Ali S , et al. A prospective, population‐based study of trends in operative vaginal delivery compared to cesarean delivery rates in low‐ and middle‐income countries, 2010‐2016. Am J Perinatol. 2019;36:730‐736. doi:10.1055/S-0038-1673656 30372772 PMC6488442

[4] Torloni MR , Opiyo N , Altieri E , et al. Interventions to reintroduce or increase assisted vaginal births: a systematic review of the literature. BMJ Open. 2023;13(2):e070640. doi:10.1136/BMJOPEN-2022-070640 PMC9930566 36787978

[5] Verma GL , Spalding JJ , Wilkinson MD , Hofmeyr GJ , Vannevel V , O'Mahony F . Instruments for assisted vaginal birth. Cochrane Database Syst Rev. 2021;9(9):CD005455. doi:10.1002/14651858.CD005455.PUB3 PMC8462579 34559884

[6] Vacca A . Handbook of Vacuum Delivery in Obstetric Practice. 3rd ed. Vacca Research; 2009.

[7] Cuerva MJ , Espinosa JÁ , Barras S , Gonzalez‐Cerron S , Ojeda F , Cortés M . Which technique is better to place a manoeuvrable vacuum extractor cup on the flexion point? Vacca vs. bird technique. J Perinat Med. 2020;48(7):694‐699. doi:10.1515/jpm-2020-0077 32692705

[8] Bahl R , Hotton E , Crofts J , Draycott T . Assisted vaginal birth in 21st century: current practice and new innovations. Am J Obstet Gynecol. 2024;230:S917‐S931. doi:10.1016/j.ajog.2022.12.305 38462263

[9] Hotton EJ , Alvarez M , Draycott TJ , et al. Outcomes of the novel Odon device in indicated operative vaginal birth. Am J Obstet Gynecol. 2021;224:607.e1‐607.e17. doi:10.1016/j.ajog.2020.12.017 PMC8192738 33316274

[10] Mottet N , Hotton E , Eckman‐Lacroix A , et al. Safety and efficacy of the OdonAssist inflatable device for assisted vaginal birth: the BESANCON ASSIST study. Am J Obstet Gynecol. 2024;230:S947‐S958. doi:10.1016/j.ajog.2023.05.016 38462265

[11] Hotton EJ , Bale N , Rose C , et al. The OdonAssist inflatable device for assisted vaginal birth—the ASSIST II study (United Kingdom). Am J Obstet Gynecol. 2024;230:S932‐S946.e3. doi:10.1016/j.ajog.2023.05.018 38462264

[12] Giacomino K , Caliesch R , Sattelmayer KM . The effectiveness of the Peyton's 4‐step teaching approach on skill acquisition of procedures in health professions education: a systematic review and meta‐analysis with integrated meta‐regression. PeerJ. 2020;8:e10129. doi:10.7717/PEERJ.10129 33083149 PMC7549471

[13] Sarker MR , Teal EN , Wiley R , et al. Contemporary trends in operative vaginal delivery and obstetric anal sphincter injuries from 2016‐2023. Pregnancy (Hoboken). 2025;1:70063. doi:10.1002/PMF2.70063 PMC12435513 40959760

[14] Skehan M . Simplifying the use of the kiwi vacuum. Int J Gynaecol Obstet. 2024;166:551‐558. doi:10.1002/IJGO.15295 38205896

[15] Skinner SM , Neil P , Murray N , et al. Clinical outcomes following implementation of an operative vaginal birth safety bundle: a prospective observational study and time‐series analysis. Am J Obstet Gynecol. 2025;233:670.e1‐670.e18. doi:10.1016/j.ajog.2025.07.013 40639779

[16] O'Brien SM , Winter C , Burden CA , Boulvain M , Draycott TJ , Crofts JF . Pressure and traction on a model fetal head and neck associated with the use of forceps, KiwiTM ventouse and the BD Odon DeviceTM in operative vaginal birth: a simulation study. BJOG. 2017;124:19‐25. doi:10.1111/1471-0528.14760 28940875 PMC7198111

[17] O'Brien SM , Winter C , Burden CA , Boulvain M , Draycott TJ , Crofts JF . Fetal head position and perineal distension associated with the use of the BD Odon DeviceTM in operative vaginal birth: a simulation study. BJOG. 2017;124:10‐18. doi:10.1111/1471-0528.14759 28940873 PMC7198112

[18] Mannella P , Giordano M , Guevara MMM , et al. Simulation training program for vacuum application to improve technical skills in vacuum‐assisted vaginal delivery. BMC Pregnancy Childbirth. 2021;21:21. doi:10.1186/S12884-021-03829-Y 33910520 PMC8082783

[19] Ennen CS , Satin AJ . Training and assessment in obstetrics: the role of simulation. Best Pract Res Clin Obstet Gynaecol. 2010;24:747‐758. doi:10.1016/j.bpobgyn.2010.03.003 20537592

