# Supplementary material for: Vacuum 5‐step technique versus OdonAssist: Which is easier to learn for professionals without experience in assisted vaginal births? A simulation study
Source: Int J Gynaecol Obstet. 2026 Jan 28;174(1):320–4. doi: 10.1002/ijgo.70836 (PMC13278629; doi:10.1002/ijgo.70836)
Supplement: Supplementary file 1 — Table S1. Linear mixed‐effects model for procedure duration at the case level. [file IJGO-174-320-s003.docx]

**Supplementary Table S1.** Linear mixed-effects model for procedure duration at the case level.

| **Predictor** | **β (SE)** | **95% CI** | **p value** |
| --- | --- | --- | --- |
| **Technique (Vacuum vs OdonAssist)** | −20.74 (22.01) | −64.34 to 22.86 | 0.348 |
| **Training order (Vacuum-first)** | −2.13 (10.74) | −23.42 to 19.15 | 0.843 |
| **Case number** | 0.03 (4.24) | −8.38 to 8.43 | 0.995 |
| **Technique × Training order** | 42.57 (14.12) | 14.59 to 70.55 | 0.003 |
| **Technique × Case number** | −0.59 (4.47) | −9.45 to 8.27 | 0.895 |
| **Occiput anterior vs transverse** | 15.37 (14.07) | −12.50 to 43.24 | 0.277 |
| **Occiput posterior vs transverse** | 13.42 (14.17) | −14.65 to 41.48 | 0.346 |
