# Supplementary material for: Vacuum 5‐step technique versus OdonAssist: Which is easier to learn for professionals without experience in assisted vaginal births? A simulation study
Source: Int J Gynaecol Obstet. 2026 Jan 28;174(1):320–4. doi: 10.1002/ijgo.70836 (PMC13278629; doi:10.1002/ijgo.70836)
Supplement: Supplementary file 2 — Table S2. Linear mixed‐effects model for perceived difficulty at the case level. [file IJGO-174-320-s001.docx]

**Supplementary Table S2.** Linear mixed-effects model for perceived difficulty at the case level.

| **Predictor** | **β (SE)** | **95% CI** | **p value** |
| --- | --- | --- | --- |
| **Technique (Vacuum vs OdonAssist)** | 0.83 (0.57) | −0.30 to 1.97 | 0.149 |
| **Training order (Vacuum-first)** | −0.01 (0.28) | −0.56 to 0.55 | 0.977 |
| **Case number** | −0.23 (0.11) | −0.45 to −0.01 | 0.037 |
| **Technique × Training order** | 0.41 (0.37) | −0.32 to 1.14 | 0.273 |
| **Technique × Case number** | 0.14 (0.12) | −0.09 to 0.37 | 0.225 |
| **Occiput anterior vs transverse** | −0.09 (0.37) | −0.81 to 0.64 | 0.814 |
| **Occiput posterior vs transverse** | −0.15 (0.37) | −0.88 to 0.59 | 0.694 |
